# Altered dynamics of scaRNA2 and scaRNA9 in response to stress correlates with disrupted nuclear organization

**DOI:** 10.1242/bio.037101

**Published:** 2018-09-03

**Authors:** Madelyn K. Logan, Marilyn F. Burke, Michael D. Hebert

**Affiliations:** Department of Cell and Molecular Biology, The University of Mississippi Medical Center, Jackson, MS 39216-4505, USA

**Keywords:** Drosha, Cajal body, Nucleolus, SMN, Coilin, WRAP53

## Abstract

Small Cajal body-specific RNAs (scaRNAs) are part of small Cajal body-specific ribonucleoproteins (scaRNPs) that modify small nuclear RNA (snRNA) in Cajal bodies (CBs). Several scaRNAs (scaRNA 2, 9 and 17) have been found to generate smaller, nucleolus-enriched fragments. We hypothesize that the fragments derived from scaRNA 2, 9 and 17 form regulatory RNPs that influence the level of modifications within rRNA by altering small nucleolar RNP (snoRNP) activity. Here we show that external factors such as DNA damaging agents can alter the scaRNA9 full length to processed fragment ratio. We also show that full-length scaRNA2 levels are likewise impacted by DNA damage, which correlates with the disruption of SMN, coilin and WRAP53 co-localization in CBs. The dynamics of scaRNA9 were also shown to be affected by Drosha levels, which suggests that this protein may participate in the biogenesis and processing of this non-coding RNA. Identification of factors that contribute to scaRNA 2, 9 and 17 processing may facilitate an assessment of how external stress can lead to changes in rRNA modifications.

## Abstract (precis)

**Summary:** Stress conditions, including DNA damaging agents, and knockdown of nuclear proteins, including SMN and Drosha, alter the processing dynamics of scaRNA 2 and 9.

## INTRODUCTION

Pre-mRNA splicing is mediated by the spliceosome, which contains small nuclear ribonucleoproteins (snRNPs) that interact with each other and pre-mRNA in order to facilitate intron removal and the joining of exons. An snRNP is comprised of small nuclear RNA (snRNA) and associated proteins. The biogenesis of spliceosomal snRNPs is complicated and involves multiple steps that take place in different cellular compartments (Kiss, 2004). For the U1, U2, U4 and U5 snRNPs, transcription of the cognate snRNA genes is conducted by RNA polymerase II and the nascent snRNAs are exported to the cytoplasm for additional processing steps that are governed by the SMN (survival of motor neuron protein) complex (Coady and Lorson, 2011; Fischer et al., 1997; Meister et al., 2002; Paushkin et al., 2002; Pellizzoni et al., 1999, 2002). One of the steps mediated by the SMN complex is the assembly of Sm proteins onto a conserved *cis* element (the Sm site) present in snRNAs. Mutations in SMN lead to most cases of spinal muscular atrophy (SMA), the leading genetic cause of infant mortality. After the cytoplasmic processing steps, the newly formed snRNPs are imported into the nucleus, where they accumulate in the Cajal body (CB), a subnuclear domain. At the CB, the snRNA component of the snRNP is modified by pseudouridylation and ribose methylation (Jady et al., 2003). These snRNA modifications are necessary for proper snRNP function and are conducted by another type of RNP known as small Cajal body-specific RNPs (scaRNPs) (Darzacq et al., 2002; Tycowski et al., 1996). In addition to U1, U2, U4 and U5, the U6 snRNP also accumulates in the CB during its biogenesis and the U6 snRNA is subjected to modification reactions mediated by scaRNPs. In contrast to the other spliceosomal snRNAs, however, U6 snRNA is generated by RNA polymerase III and the U6 snRNP has biogenesis steps that take place in the nucleolus (not the cytoplasm) before accumulating in CBs (Kunkel et al., 1986). After additional maturation steps (binding of snRNP-specific proteins and U4/U6 assembly), the snRNPs are ready to leave the CB and perform their splicing activity.

The biogenesis of scaRNPs is not as clearly understood as that for snRNPs. There are three different classes of scaRNPs, defined by conserved motifs present in the scaRNA: box C/D, box H/ACA and mixed domain scaRNAs that have both box C/D and box H/ACA elements. These different types of scaRNAs base pair with target snRNA and guide the activity of enzymes present in the scaRNP complex to modify specific sites within the snRNA (Kiss, 2004; Yu et al., 1998). Box C/D scaRNPs contain fibrillarin, which conducts ribose methylation, and box H/ACA scaRNPs contain dyskerin, which conducts pseudouridylation (Baserga et al., 1991; Fatica et al., 2000; Gautier et al., 1997; Schimmang et al., 1989; Szewczak et al., 2002; Tyc and Steitz, 1989; Watkins et al., 1996). Mixed domain scaRNPs contain both fibrillarin and dyskerin. In addition to fibrillarin and dyskerin, scaRNPs contain additional accessory proteins. Interestingly, scaRNAs containing H/ACA motifs also contain a *cis* element known as the CAB box. The CAB box is a Cajal body localization signal and interacts with the scaRNP-biogenesis factor WRAP53 (Richard et al., 2003). A CAB box is also present in telomerase RNA, and interactions between WRAP53 and telomerase RNA contribute to telomerase maturation steps that occur in the CB (Jady et al., 2004; Mahmoudi et al., 2010; Tycowski et al., 2009; Venteicher et al., 2009; Zhu et al., 2004). Unlike box H/ACA scaRNAs, a CAB motif is not present in box C/D scaRNAs, so it is unclear how these RNAs are targeted to the CB (Marnef et al., 2014). It is possible that interactions between WRAP53 and a *cis* element known as the G.U/U.G wobble stem which is present in some box C/D scaRNAs, such as scaRNA 7 and 28, allows for the accumulation of these box C/D scaRNAs in CBs (Marnef et al., 2014). However, other box C/D scaRNAs do not contain the G.U/U.G wobble stem, and it is possible that interactions between these scaRNAs and proteins enriched in the CB such as coilin contribute to the accumulation of these scaRNAs in CBs (Poole et al., 2016, 2017).

The majority of scaRNAs are derived from introns, although a few (scaRNA2, scaRNA17 and telomerase RNA) are independently transcribed. Curiously, three scaRNAs (scaRNA2, scaRNA9 and scaRNA17) have been shown to undergo processing steps that can produce smaller, nucleolus-enriched fragments (Tycowski et al., 2004). The function of the fragments derived from scaRNA 2, 9 and 17 are unknown, but we have recently provided evidence that they may form a new class of RNP, the regulatory RNP (Poole et al., 2017). Regulatory RNPs are predicted to alter the level of modifications within rRNA by altering small nucleolar RNP (snoRNP) activity. As found for snRNA, human rRNA is extensively modified and contains approximately 100 each of pseudouridine and ribosome methylation modifications, most of which are mediated by snoRNPs (Darzacq et al., 2002; Khan and Maden, 1978; Maden et al., 1972; Maden and Salim, 1974). There are two kinds of snoRNPs: box H/ACA which contain dyskerin and are responsible for the pseudouridylation of rRNA, and box C/D which contain fibrillarin and perform ribosome methylation of rRNA (Kiss, 2004). Base pairing between the snoRNA component of the snoRNP with rRNA guides modification reactions. We hypothesize that interactions between the RNA component of regulatory RNPs with snoRNA may alter snoRNP activity and thus impact rRNA modification (Poole et al., 2017). Our recent work shows that regulatory RNPs may impact the activity snord16, snord68, snord111 and snord94, thereby influencing the modification of 18S rRNA (at positions U428 and A484), 28S rRNA (at positions A2388 and G3923) and U6 snRNA (at position C62) (Burke et al., 2018; Poole et al., 2017).

Given the potential role of fragments derived from scaRNA 2, 9 and 17 in the regulation of rRNA modification, it is imperative that we understand the signaling cues and mechanisms that govern the processing of full-length scaRNA 2, 9 and 17 (which accumulate in the CB) to fragments (which accumulate in the nucleolus). Our previous work implicates the CB proteins coilin, SMN, WRAP53 and the product of the *COILP1* pseudogene, coilp1, as factors that contribute to scaRNA 2, 9 and 17 processing (Poole et al., 2016, 2017). However, there have not been any studies that seek to determine the environmental conditions, such as in response to stress, that impact the processing of these three scaRNAs. Such knowledge may indicate how stress conditions alter rRNA modification which in turn may lead to the changes in the translation of mRNAs that produce proteins that deal with the stress. In this work, we examined the dynamics of scaRNA 2 and 9 in different stress conditions. We also observed that disruptions in nuclear organization, specifically in the composition of the CB, is correlated with altered levels of scaRNA 2 and 9. Notably, we observed that SMN accumulation in the CB is important for the processing of scaRNA9, and the reduction of SMN decreases the processing of scaRNA9. Lastly, we have found that scaRNA9 dynamics are altered upon Drosha reduction, which suggests that the ratio of the full length scaRNA9 to its processed fragment is influenced by this protein.

## RESULTS

**Fig. 1.**
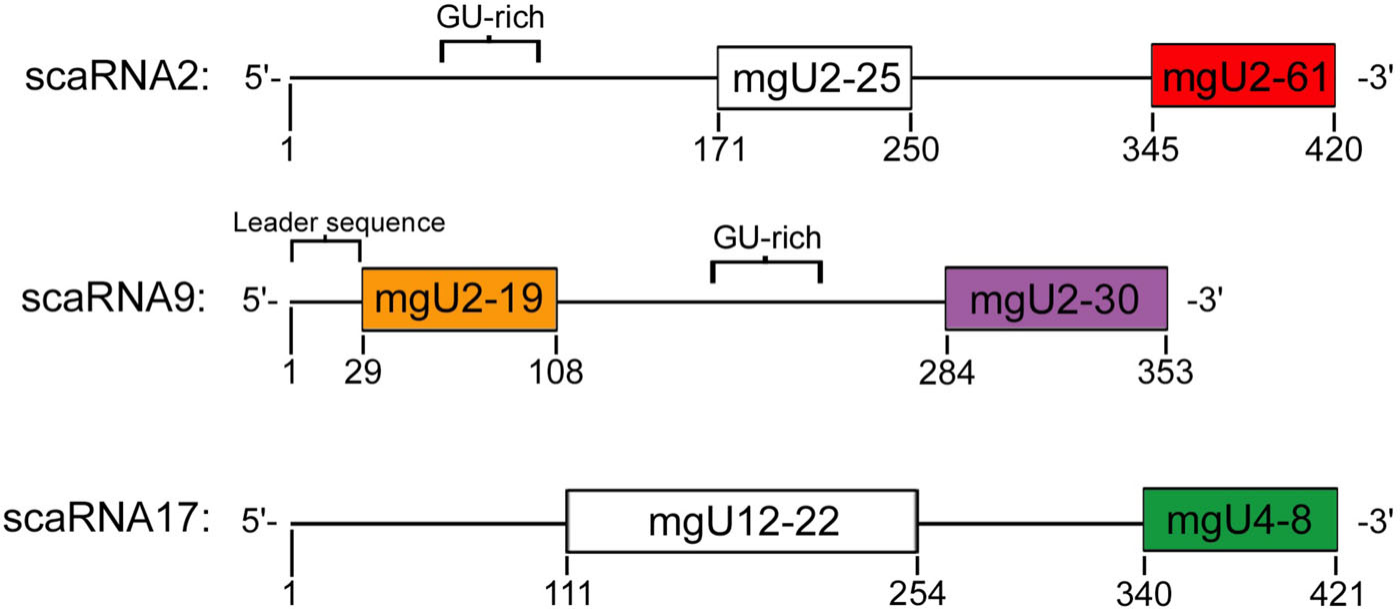
**Schematic representation of scaRNA 2, 9 and 17.** scaRNA 2 and 17 are derived from independently transcribed genes, while scaRNA9 is encoded within the intron of its host gene, *CEP295*. Guide domains are shown for each scaRNA and fragments generated from each scaRNA are colored. The nomenclature of the guide domains is as follows: mgU2-25 is **m**ethylation **g**uide for the modification of U2 snRNA at position 25. The GU-rich repeat region in scaRNA2 and 9 is indicated (Enwerem et al., 2015; Poole et al., 2016). Also shown is the leader sequence at the 5′ end of scaRNA9 (Poole et al., 2017; Tycowski et al., 2004).

ScaRNA 2 and 17 are derived from independently transcribed genes, while scaRNA9 is encoded within the intron of its host gene (Tycowski et al., 2004). Full-length scaRNA2, scaRNA9 and scaRNA17 contain domains that participate in the ribose methylation of specific sites within U2, U12 and U4 snRNA (Fig. 1). For example, scaRNA2 contains the mgU2-25 domain which base pairs with U2 snRNA and serves as a methylation guide for position 25 of U2 snRNA. These full-length scaRNAs can also be internally processed, generating fragments (colored in Fig. 1) that are enriched in the nucleolus. Our previous work indicates that these fragments may become regulatory RNPs that influence rRNA modification via interactions with snoRNPs (Poole et al., 2017). Little is known about how scaRNA 2, 9 and 17 are processed, but we have identified *cis* elements, the GU-rich region (in scaRNA2 and scaRNA9) and the leader sequence of scaRNA9 (denoted in Fig. 1) that impact the processing of these scaRNAs (Enwerem et al., 2015; Poole et al., 2016).

### The dynamics of ectopically expressed scaRNA9 are influenced by various treatments

**Fig. 2.**
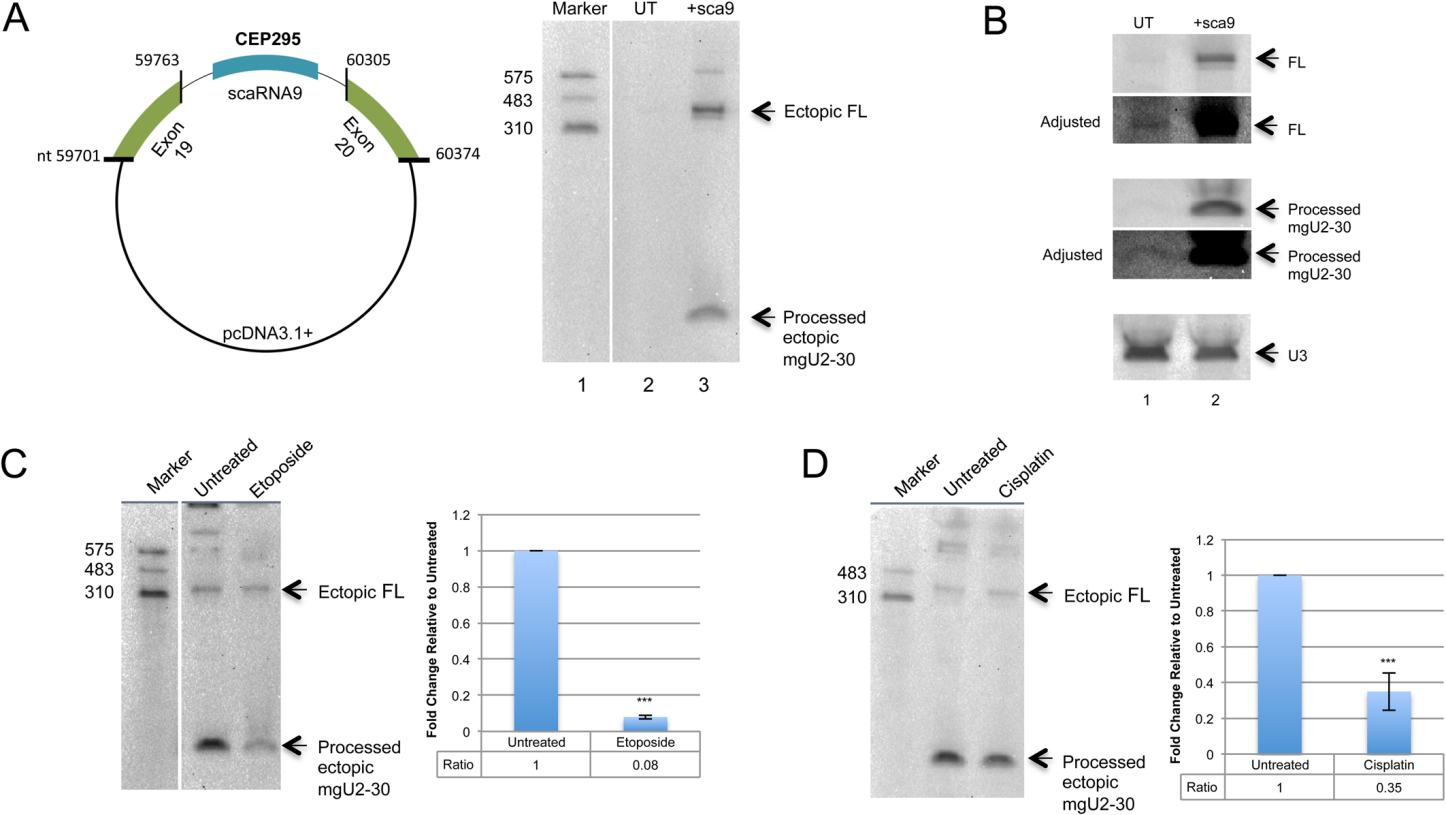
**Etoposide and cisplatin alter the dynamics of full-length scaRNA9 and the mgU2-30 fragment.** (A) Left, schematic of the plasmid used for scaRNA9 expression from the *CEP295* host gene. Right, 10 µg of RNA from untransfected (UT) or scaRNA9 transfected cells was subjected to electrophoresis, northern blotting, and detection with a 5′ DIG probe to scaRNA9. Endogenous full-length scaRNA9 (lane 2) is only slightly visible compared to ectopic full-length (FL) scaRNA9 (lane 3). The mgU2-30 processed fragment is easily detected in RNA isolated from cells transfected with scaRNA9 (lane 3). (B) Northern blot showing endogenous and ectopic scaRNA9 FL and mgU2-30 fragment. 10 µg of RNA from untransfected (UT) or scaRNA9 transfected cells was subjected to electrophoresis, northern blotting, and detection with a 5′ and 3′ labeled DIG probe to scaRNA9. Endogenous FL scaRNA9 signal (lane 1) is very faint compared to that obtained in scaRNA9 expressing RNA (lane 2). An adjusted image is shown to more easily identify endogenous FL scaRNA9. Likewise, the mgU2-30 fragment is easily detectable in RNA from cells expressing scaRNA9, endogenous mgU2-30 is more difficult to detect. An adjusted image is shown to more easily identify endogenous mgU2-30. The same membrane was reprobed for U3 snoRNA (snord3) to verify that approximately equal amounts of RNA was loaded in each lane (lower panel). (C,D) HeLa cells were transfected with scaRNA9 pcDNA3.1+ for 24 h. 7.5 μM etoposide (C) or 3 μg/ml cisplatin (D). Treatments occurred 7 h after transfection. scaRNA9 was detected after northern blot detection using a 5′ DIG labeled probe. Histograms were generated from the quantified images by normalizing the processed mgU2-30 signal to the full-length scaRNA9 signal. The data for the treated samples was then normalized to the fragment/full-length ratio obtained for untreated RNA. ***P-value <0.0005, error bars represent standard deviation. *N*=5 for etoposide treatments and *n*=7 for cisplatin treatments with n representing biological repeats.

To begin our analysis into conditions that alter the processing of scaRNA 2, 9 and 17, we first examined the level of the mgU2-30 fragment (Fig. 1) derived from an ectopically expressed scaRNA9. Ectopically expressed scaRNA9 in the context of its *CEP295* host gene generates a full-length scaRNA9 and processed mgU2-30 fragment that are indistinguishable in size to the endogenous scaRNA9 and mgU2-30 fragment, but much easier to detect (Fig. 2A,B). In Fig. 2A, cells were untransfected (UT) or transfected with pcDNA3.1+ expressing scaRNA9 in the context of its host gene. 10 µg of isolated RNA was then subjected to electrophoresis and northern blotting, followed by detection of endogenous and ectopic scaRNA using a 5′ Digoxigenin (DIG) labeled probe. Full-length scaRNA9 and the mgU2-30 fragment are easily detected in RNA from cells transfected with pcDNA3.1+ harboring scaRNA9 (Fig. 2A, lane 3) using this probe. In contrast, endogenous full-length scaRNA9 is only faintly detected, and the mgU2-30 fragment is not observed, when using the 5′ DIG labeled probe (Fig. 2A, lane 2). To detect the endogenous mgU2-30 fragment, we employed a probe that is DIG labeled on both the 5′ and 3′ ends. 10 µg of RNA from untransfected cells or RNA from cells transfected with scaRNA9 plasmid was evaluated by northern blotting and detection with the 5′ and 3′ DIG labeled probe (Fig. 2B). As shown in lane 1, endogenous full-length scaRNA9 and mgU2-30 fragment are detected, and are the same size as that found for RNA obtained from cells ectopically expressing scaRNA9 (lane 2). Adjusted images are provided in order to more easily visualize endogenous full-length scaRNA9 and the mgU2-30 fragment. The blot was then reprobed for U3 snoRNA (snord3) in order to verify that approximately equal amounts of RNA were loaded in each lane (lower panel). The data provided in Fig. 2A and B thus show that ectopically expressed scaRNA9 gives rise to full-length scaRNA9 and mgU2-30 fragment that are the same size as that found for the endogenous versions. Therefore, we decided to utilize ectopically expressed scaRNA9 as a model system to examine treatments and conditions that may alter the processing of scaRNA 2, 9 and 17, with the caveat that scaRNA2 and scaRNA17 may be subject to other processing controls.

Although we are specifically interested in scaRNA 2, 9 and 17 processing dynamics, the experiments shown here do not formally exclude contributions by factors that alter the degradation of the full-length scaRNA and derived processed fragment, or influence the half-life of these RNAs. Hence by examining the ratio of full-length scaRNA to its processed fragment on a northern blot, we are observing the end result of a balance between biogenesis, processing and degradation components that collectively regulate the amount of full-length scaRNA and its processed fragment. To begin this analysis, HeLa cells were transfected with pcDNA3.1+ containing scaRNA9 followed by treatment with etoposide or cisplatin, which are commonly used DNA damaging agents (Cheung-Ong et al., 2013). RNA isolated from these cells was then subjected to northern blotting using a 5′ DIG labeled probe which detects full-length scaRNA9 as well as the processed mgU2-30 fragment (the same probe was used in Fig. 2A). As shown in Fig. 2C and D, both etoposide and cisplatin treatments significantly decrease the amount of the ectopic processed mgU2-30 fragment relative to the amount of ectopic full-length scaRNA9 when compared to untreated.

**Fig. 3.**
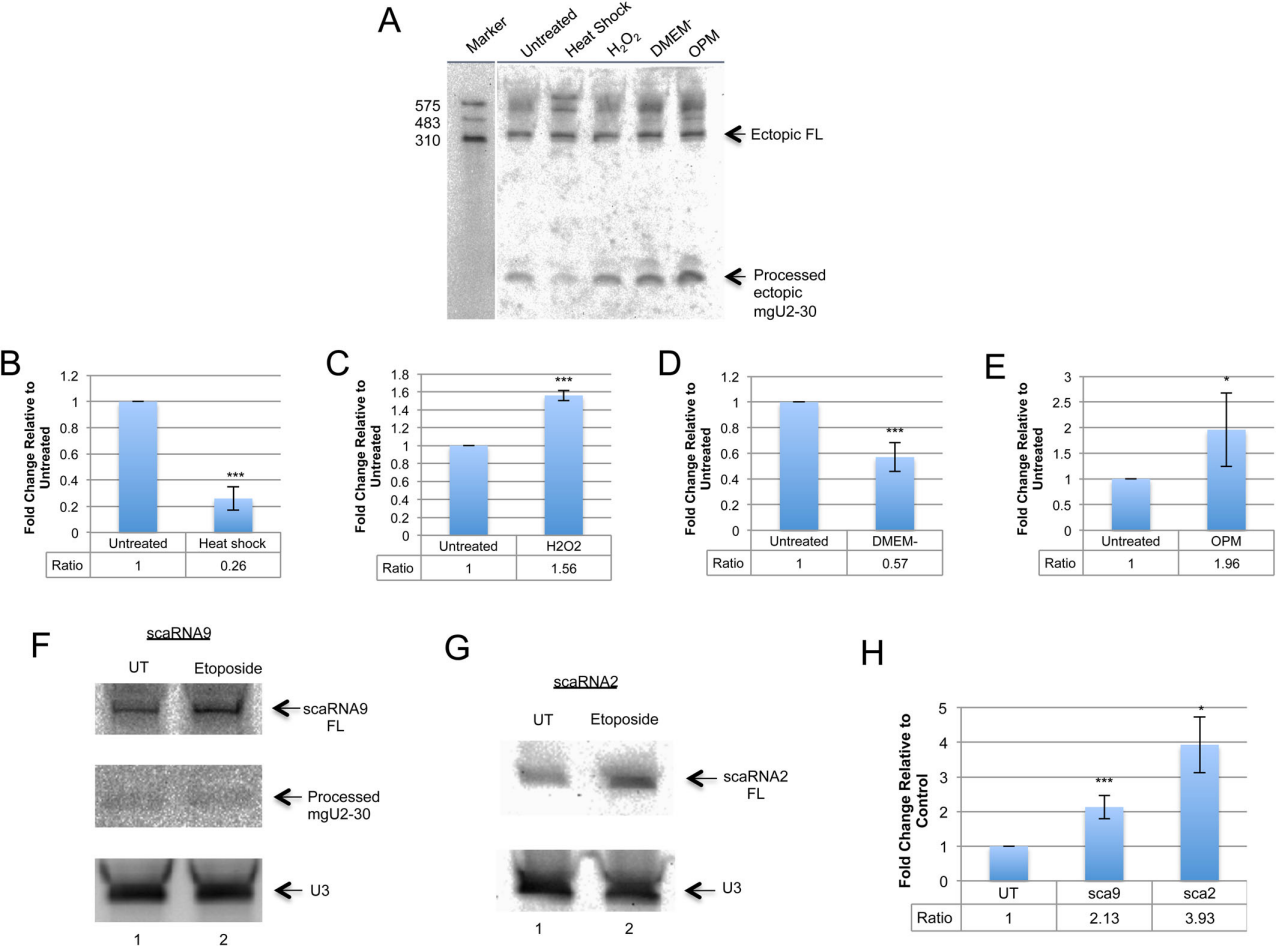
**Stress conditions differentially affect the dynamics of ectopic scaRNA9 and endogenous scaRNA 9 and 2.** (A) HeLa cells were transfected with scaRNA9 pcDNA3.1+ for 24 h and treatments occurred 7 h after initial transfection: 42°C incubation (heat shock) *n*=6, 0.2 mM H_2_O_2_ (oxidative stress) *n*=6, DMEM lacking serum (DMEM^−^) *n*=7, or Optimem reduced serum medium (OPM) *n*=7. scaRNA9 was detected after northern transfer using a mixture of a 5′ DIG labeled and 3′ DIG labeled probe. (B-E) Histograms were generated after using the volume analyze tool on the adjusted image to quantify the ratio of the processed mgU2-30 fragment to the full-length scaRNA9. The treated condition was then normalized to the untreated. *P-value <0.05; ***P-value <0.0005, error bars represent standard error. (F,G) HeLa cells were treated with 17 μM etoposide for 24 h. Northern blots using probes for scaRNA9 (F) or scaRNA2 (G) show etoposide treated cells (lane 2) have an increase in full-length signal compared to untreated (lane 1). (H) A histogram was generated by normalizing the full-length signal to the snoRNA U3 signal. *N*=14 for scaRNA9 data and *n*=5 for scaRNA2 data (*P-value <0.05; ***P-value <0.0005, error bars represent standard error). In all cases ‘*n*’ equals biological repeats.

We next investigated the impact of four additional treatments on the full-length to processed fragment ratio of ectopically expressed scaRNA9. These four treatments were heat shock, oxidative stress induced by H_2_O_2_, serum starvation (DMEM^−^) and serum reduction (OPM). Since previous reports have shown that serum starvation disrupts canonical CBs (Andrade et al., 1993), we also wanted to explore the consequence of serum reduction using a reduced serum medium (OptiMem). As shown in Fig. 3A, and quantified in Fig. 3B, the amount of ectopic mgU2-30 fragment relative to full-length ectopic scaRNA9 is decreased upon heat shock treatment compared to that found in untreated cells. A reduction in the relative amount of mgU2-30 was also observed upon serum starvation (Fig. 3A, quantified in Fig. 3D). In contrast, H_2_O_2_ treatment and serum reduction (OPM) resulted in an increase in the amount of the mgU2-30 fragment relative to full-length scaRNA9 compared to untreated (Fig. 3A, quantified in Fig. 3C,E). At present we do not understand why reduced serum increases the relative amount of mgU2-30 while serum starvation decreases the amount of this fragment. Collectively, these data support the hypothesis that the dynamics of scaRNA9 in terms of the relative amount of full-length and processed fragment may be modified by environmental conditions.

### Endogenous scaRNA9 and scaRNA2 dynamics are altered by etoposide treatment

In order to demonstrate that the ratio of mgU2-30 fragment to full-length scaRNA9 is altered for endogenous scaRNA9 as observed for ectopically expressed scaRNA9 (Figs 2 and 3A), untransfected cells were untreated or treated with etoposide (17 µM for 24 h) and isolated RNA was subjected to northern blotting and detection (Fig. 3F). To observe the endogenous mgU2-30 fragment, the 5′ and 3′ DIG labeled probe used in Fig. 2B was employed. As shown in Fig. 3F (upper panel), the amount of endogenous full-length scaRNA9 is increased upon etoposide treatment (lane 2) compared to untreated (UT, lane 1). However, the amount of endogenous mgU2-30 fragment is not impacted by etoposide (Fig. 3F, middle panel). To quantify the increase in full-length scaRNA9 observed with etoposide treatment, the same blot was reprobed for U3 snoRNA (Fig. 3F, lower panel) and the full-length scaRNA9 signal was normalized to the U3 snoRNA signal. This quantification is shown in Fig. 3H, and demonstrates that etoposide treatment significantly increases the relative amount of endogenous full-length scaRNA9 by 2.1-fold compared to that found in untreated cells.

We then evaluated if etoposide treatment alters the dynamics of scaRNA2. For this work, RNA isolated from untreated (UT) or etoposide-treated cells was subjected to northern blotting and detection with a 5′ DIG labeled probe that detects scaRNA2 (Fig. 3G). This probe is not sensitive enough to detect the endogenous scaRNA2 processed fragment, mgU2-61, with the amount of RNA used here. As shown in Fig. 3G, endogenous full-length scaRNA2 is increased upon etoposide treatment (lane 2) compared to that obtained from untreated cells (lane 1). The blot was then reprobed to detect U3 snoRNA (lower panel), and this U3 signal was used to normalize the scaRNA2 data for quantification (shown in Fig. 3H). As found for endogenous scaRNA9, full-length endogenous scaRNA2 levels are significantly increased (3.9-fold) compared to those obtained in untreated RNA (Fig. 3H). Collectively, the data shown in Figs 2 and 3 support the hypothesis that scaRNA 2 and 9 may be subjected to controls that regulate the dynamics of the full-length scaRNA and the processed fragments derived from them. In particular, certain treatments, such as etoposide, may inhibit the generation of processed fragments from full-length scaRNA 2 and 9.

### Disrupted nuclear organization upon etoposide and cisplatin exposure

**Fig. 4.**
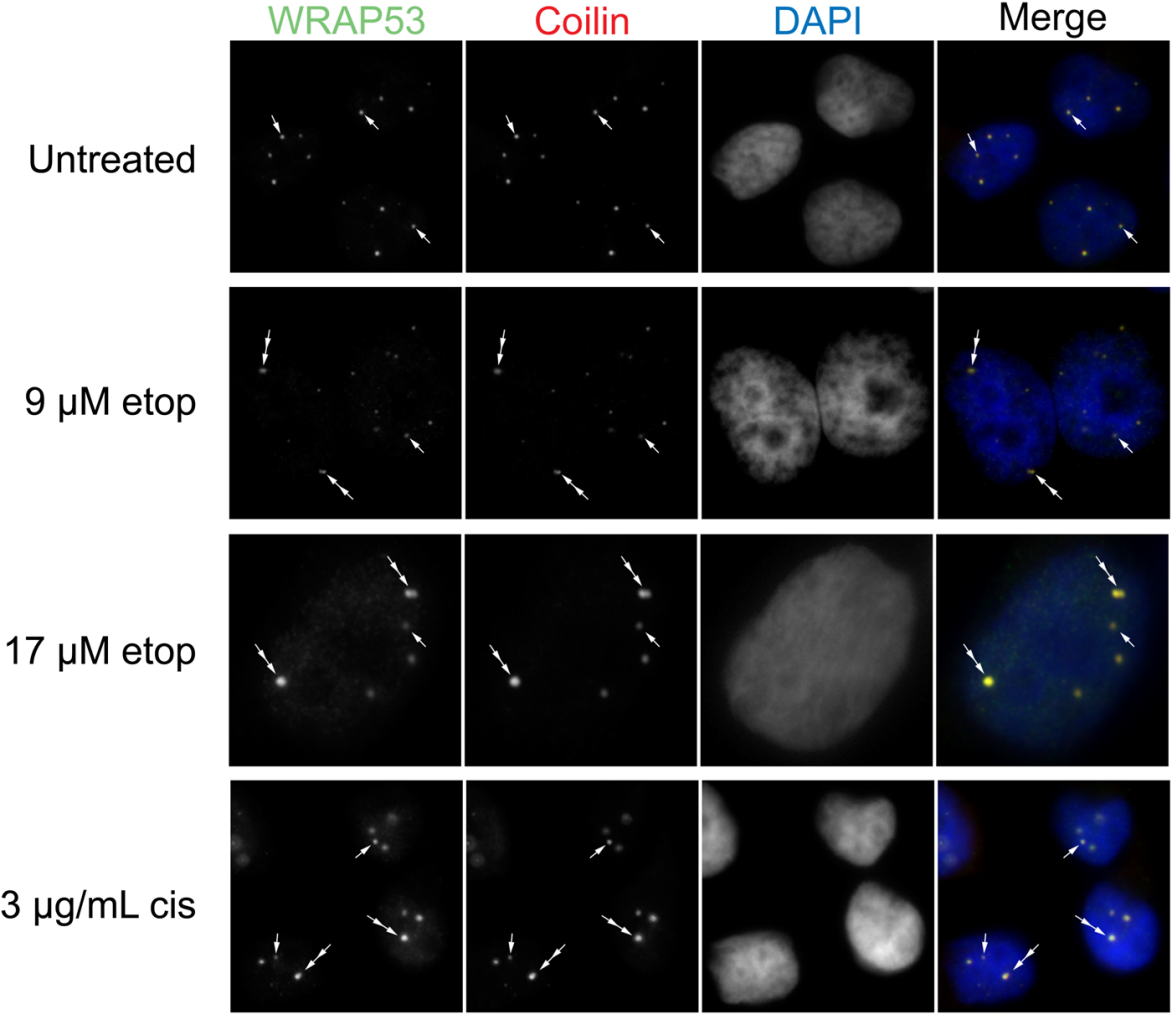
**Etoposide or cisplatin treatment disrupt CBs.** HeLa cells were treated with etoposide or cisplatin, followed by fixation and detection of WRAP53 (green) and coilin (red). DAPI was used to stain the nucleus (blue). CBs are represented by arrows. Double arrows demarcate coilin foci with altered morphology/composition.

**Fig. 5.**
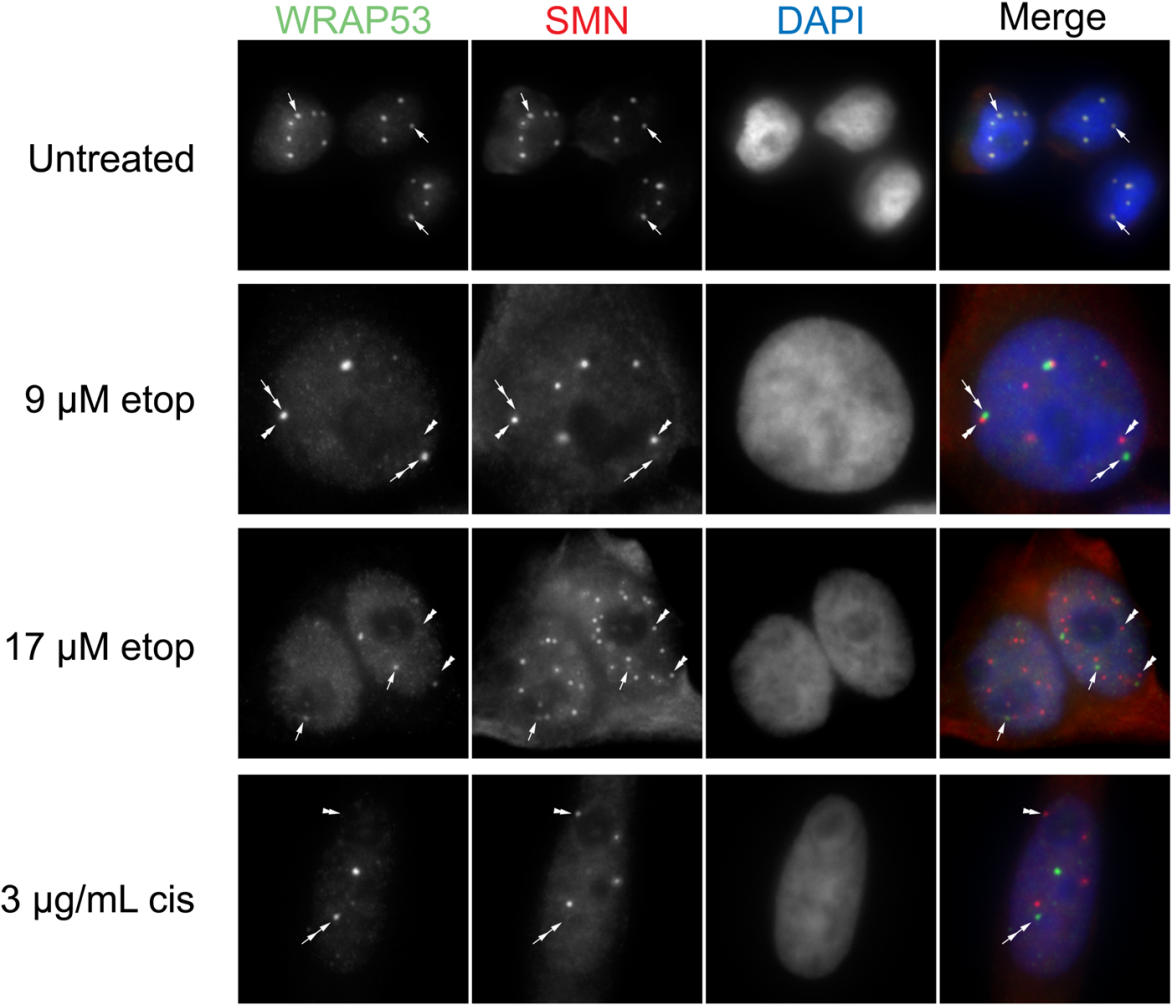
**Etoposide or cisplatin treatment in HeLa cells promotes the shift of SMN out of CBs.** HeLa cells were treated with etoposide or cisplatin, followed by fixation and detection of WRAP53 (green) and SMN (red). DAPI was used to stain the nucleus (blue). CBs are represented by arrows. Double arrowheads represent gems. Double arrows indicate WRAP53 foci with low levels of SMN.

**Fig. 6.**
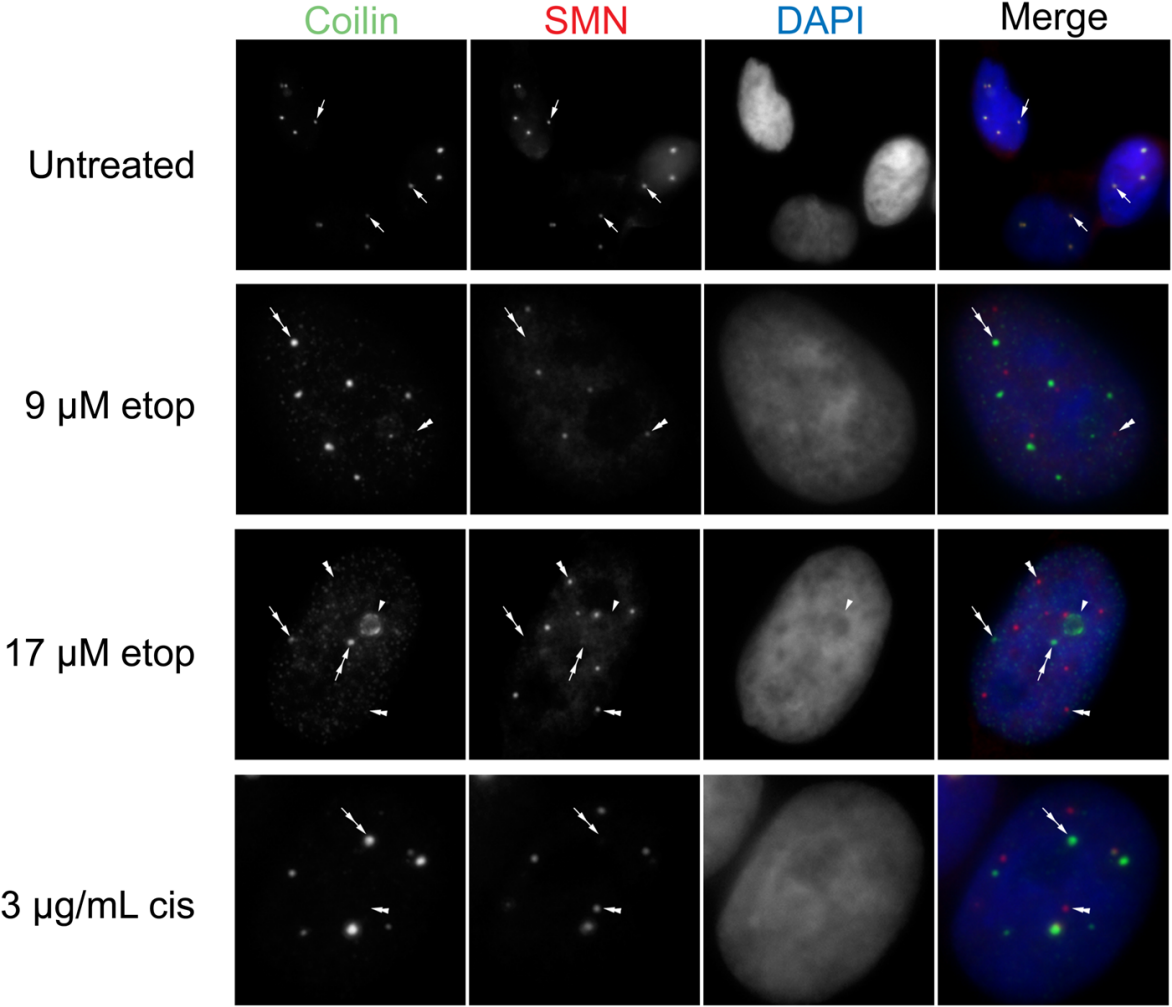
**Etoposide or cisplatin treatment induces gems.** HeLa cells were treated with etoposide or cisplatin, followed by fixation and detection of coilin (green) and SMN (red). DAPI was used to stain the nucleus (blue). CBs are represented by arrows. Double arrowheads represent gems. Single arrowheads represent nucleolar accumulation of coilin. Double arrows indicate coilin foci with low levels of SMN.

Our previously published work strongly indicates that proteins enriched within the CB (coilin, WRAP53 and SMN) contribute towards scaRNA 2, 9 and 17 processing (Enwerem et al., 2014, 2015; Poole et al., 2016, 2017). Specifically, we have observed that coilin reduction correlates with the increased production of the mgU2-61 fragment derived from scaRNA2 (Enwerem et al., 2015). In contrast, SMN reduction correlates with decreased scaRNA2 processing (Enwerem et al., 2015). Since the data we have presented in Figs 2 and 3 show that the dynamics of scaRNA9 and scaRNA2 are altered upon etoposide and cisplatin treatment, we speculated that these stress conditions may be disrupting the nuclear organization of coilin, SMN and WRAP53. We therefore examined the localization of these proteins in untreated, etoposide (9 and 17 µM) and cisplatin (3 µg/ml) treated HeLa cells (Figs 4–6). In agreement with previous reports that have examined coilin localization in the presence of these DNA damaging agents (Broome and Hebert, 2013; Gilder et al., 2011; Song et al., 2017; Velma et al., 2012), we observed alterations in the localization of coilin and the composition and morphology of CBs. As shown in Figs 4–6, normal CBs are indicated by arrows, and they contain coilin, WRAP53 and SMN. Cultures treated with etoposide or cisplatin have fewer cells with canonical CBs, and instead have coilin foci with altered morphology and lack SMN (double arrows) or have coilin accumulations in the nucleolus (arrowhead). Note that previous reports have reported that coilin accumulations are often only observed in one nucleolus, as determined by co-staining with Nopp140 or fibrillarin, and not every cell has nucleolar accumulations (Gilder et al., 2011). In addition to these changes, we also observe an increase in the percent of cells that contain SMN foci (gems, indicated by double arrowhead) upon etoposide or cisplatin treatment.

Interestingly, in the nucleus WRAP53 is co-localized with coilin in the CB and continues to associate with coilin despite the induction of nuclear disruption by DNA damaging agents (Fig. 4, double arrow). In contrast, nuclear SMN, which in untreated cells co-localizes with WRAP53 and coilin in the CB, forms structures separate from WRAP53 (Fig. 5) or coilin (Fig. 6), most likely gems (double arrowheads), upon treatment with etoposide or cisplatin. Therefore, the association of coilin, WRAP53 and SMN that is normally observed in untreated cells (arrows) is disrupted upon etoposide and cisplatin treatment, with SMN being displaced from the coilin/WRAP53 complex (double arrows) and forming gems (double arrowheads). These findings suggest that SMN displacement from coilin and WRAP53 upon DNA damage correlates with a reduction in the amount of the mgU2-30 fragment derived from scaRNA9.

### SMN reduction decreases the relative amount of the mgU2-30 fragment

**Fig. 7.**
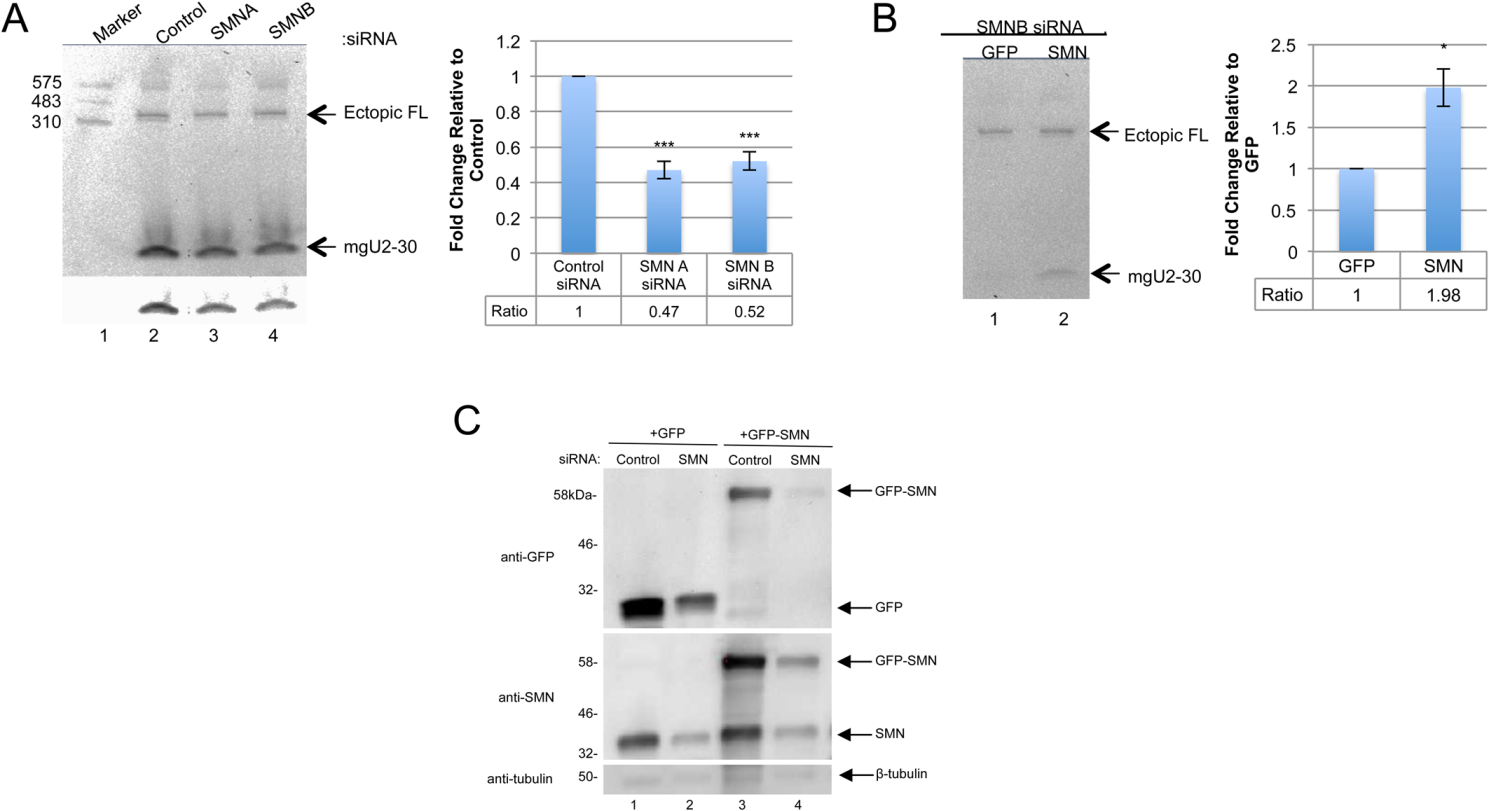
**Reduction of SMN decreases the relative amount of the mgU2-30 fragment from ectopically expressed scaRNA9.** (A) Northern blot of RNA from HeLa cells transfected with control (lane 2), SMN-A (lane 3) or SMN-B (lane 4) siRNA for 24 h and then transfected with pcDNA 3.1+ scaRNA9 for 24 h. A histogram was created quantifying the ratio between the processed mgU2-30 fragment and ectopic full-length from six different biological repeats. (B) Northern blot of RNA from HeLa cells transfected with SMN-B siRNA for 24 h followed by the co-transfection of scaRNA9 and GFP plasmid (lane 1) or scaRNA9 and GFP-SMN plasmid (lane 2) for 24 h. A histogram was created quantifying the ratio between mgU2-30 and ectopic full-length from eight different biological repeats. (C) Western blot showing SMN knockdown and rescue. HeLa cells were transfected with negative control or SMN-B siRNA for 24 h and then co-transfected with GFP plasmid (lanes 1 and 2) or with GFP-SMN plasmid (lanes 3 and 4) for 24 h. The membrane was then probed for GFP and SMN, respectively. β-tubulin was then probed as a loading control. Standard error was used to generate error bars. Student’s *t*-test was used to determine statistical significance, indicated by * corresponding to a *P*-value<0.05 (B); *** corresponding to a *P*-value<0.0005 (A).

Given that the disruption of SMN from the coilin/WRAP53 complex upon etoposide or cisplatin treatment (Figs 4–6) correlates with an altered full-length to processed fragment ratio of ectopically expressed scaRNA9 in these same conditions (Fig. 2C,D), we next examined if SMN reduction would decrease the relative level of the scaRNA9-derived mgU2-30 fragment. For this work, cells were treated with control siRNA or two different siRNAs targeting SMN mRNA (SMNA, SMNB) for 24 h. After 24 h, cells were then transfected with a plasmid expressing scaRNA9 and this transfection was allowed to continue for another 24 h before cells were harvested and RNA was isolated. Hence, the siRNA-mediated knockdown of SMN was for 48 h and plasmid expression of scaRNA9 was for 24 h. Northern blotting and detection shows that SMN reduction mediated by SMNA or SMNB siRNA decreases the amount of the ectopic mgU2-30 fragment relative to ectopic full-length scaRNA9 when compared to cells treated with control siRNA (Fig. 7A, quantification shown in histogram). The lower panel of Fig. 7A shows an adjusted image of the mgU2-30 region which more clearly shows that SMN knockdown (lanes 3 and 4) decreases the amount of this fragment compared to control siRNA (lane 2). We next examined if we could rescue this decreased fragment phenotype in the SMN knockdown background by ectopically expressing SMN. For this experiment, cells were treated with SMNB siRNA for 24 h, followed by the co-transfection of plasmids expressing scaRNA9 and GFP alone or scaRNA9 with GFP-SMN. GFP-SMN has been shown to properly localize to the cytoplasm and CBs, and associate with known interactors of SMN (Shpargel and Matera, 2005). After an additional 24 h, cells were harvested and isolated RNA was subjected to northern blotting and detection of full-length scaRNA9 and the mgU2-30 fragment. As shown in Fig. 7B, more mgU2-30 fragment is detected in cells expressing GFP-SMN compared to that observed in cells with GFP alone expression (histogram).

For these experiments, we have intentionally used a GFP-SMN construct that can be targeted by SMN siRNA in order to not dramatically increase SMN levels. A western blot of lysate obtained from control or SMN siRNA cells transfected with GFP or GFP-SMN is shown in Fig. 7C. As shown in Fig. 7C, detection of SMN using an anti-SMN antibody demonstrates that SMN is reduced upon SMN siRNA treatment compared to control siRNA (middle panel, compare SMN signal in lane 2 to that in lane 1, and SMN signal in lane 4 to that in lane 3). However, SMN knockdown cells transfected with GFP-SMN contain approximately twofold more SMN (SMN plus GFP-SMN) compared to SMN knockdown cells transfected with GFP alone (middle panel, compare the SMN plus GFP-SMN signal in lane 4 to the SMN signal in lane 2). The upper panel of Fig. 7C is the same membrane probed with anti-GFP, showing the expression of GFP alone (lanes 1 and 2) and GFP-SMN (lanes 3 and 4) in control or SMN siRNA backgrounds. The bottom panel shows the detection of anti-beta tubulin as a loading control. In summary, therefore, the relative amount of the mgU2-30 fragment is increased in the SMN knockdown background upon the addition of GFP-SMN compared to that observed with GFP alone (Fig. 7B). Analysis of the total amount of SMN in these experiments, which consists of GFP-SMN plus endogenous SMN (Fig. 7C, lane 4), demonstrates that these results are not the result of highly overexpressed SMN.

### Drosha reduction alters the full-length to processed fragment ratio of ectopic and endogenous scaRNA9

**Fig. 8.**
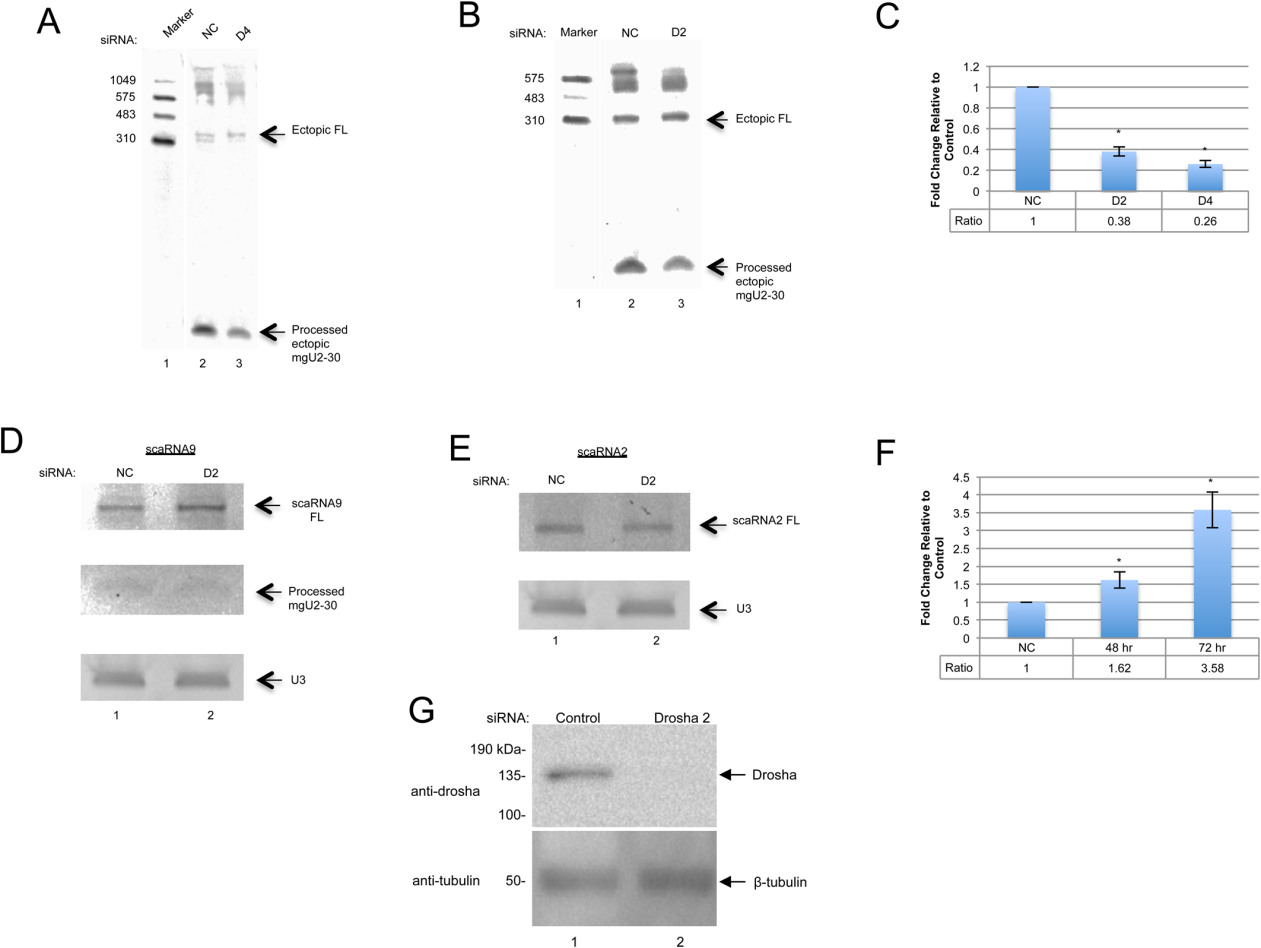
**Identification of Drosha as a factor that impacts on scaRNA 2 and 9 dynamics.** (A-C) Northern blots of RNA from HeLa cells transfected with negative control (A,B, lane 2), Drosha 4 (A, lane 3), or Drosha 2 (B, lane 3) siRNA for 24 h and then transfected with pcDNA 3.1+ scaRNA9 for 24 h. A 5′ DIG probe to scaRNA9 was used. A histogram (C) was generated from the quantified images by normalizing the processed mgU2-30 signal to the full-length scaRNA9 signal. Data for the knockdown samples was then normalized to the fragment/full-length ratio obtained for control RNA. Standard error was used to generate error bars. Student’s *t*-test was used to determine statistical significance, indicated by * corresponding to a *P*-value <0.05. *N*=25 for control treatments, *n*=21 for D2 treatments and *n*=6 for D4 treatments. (D-F) Northern blots of endogenous RNA from HeLa cells transfected with negative control (lane 1) or Drosha 2 (lane 2), siRNA for 72 h and then probed for scaRNA9 (D) or scaRNA2 (E). A histogram (F) was generated for the scaRNA9 data by normalizing the full-length signal to the snoRNA U3 signal. *N*=7 for 48 h treatments (not shown) and *n*=5 for 72 h treatments. (G) Western blot showing Drosha knockdown. HeLa cells were transfected with negative control or Drosha 2 siRNA for 48 h (lanes 1 and 2). The membrane was then probed with Drosha antibody. β-tubulin was probed lastly as a control. In all cases ‘*n*’ equals biological repeats.

We have identified several factors that may contribute to scaRNA 2, 9 and 17 processing (Poole et al., 2016, 2017). Since scaRNA 2, 9 and 17 are predicted to fold into stem-loop structures (Tycowski et al., 2004), we next explored if scaRNA 2, 9 and 17 may be unorthodox substrates for Drosha, which is a member of the RNase III family that initiates microRNA processing (Denli et al., 2004; Lee et al., 2003; Zeng et al., 2005). In the nucleus, Drosha enzymatically cleaves primary-miRNA (pri-miRNA) into the stem and loop structure of pre-miRNA that will go on to the cytoplasm to be processed again by Dicer (Bernstein et al., 2001; Grishok et al., 2001; Hutvagner et al., 2001; Ketting et al., 2001; Knight and Bass, 2001). Interestingly, the components of RNA interference, including Dicer, are present and active in the nucleus (Gagnon et al., 2014). We therefore hypothesized that reducing a preliminary step in miRNA formation by knocking down Drosha would alter the dynamics, and possibly the processing, of scaRNA 2, 9 and 17. For this work, we used two different siRNAs targeting Drosha (D2 and D4), both of which effectively reduce Drosha protein levels (Fig. 8G). We first examined the full-length and mgU2-30 fragment levels of ectopically expressed scaRNA9 in the Drosha knockdown background. Cells were treated with control or Drosha siRNA for 24 h, followed by transfection with pcDNA3.1+ expressing scaRNA9. 24 h later, cells were harvested and RNA was isolated. Thus for these experiments the Drosha knockdown is 48 h and DNA transfection is for 24 h. RNA was subjected to northern blotting and scaRNA9 and mgU2-30 fragment were detected with a 5′ DIG labeled probe. Both Drosha siRNAs result in a relative decrease in the amount of the mgU2-30 fragment relative to full-length scaRNA9 compared to that found when using control siRNA (Fig. 8A, Drosha4, D4, siRNA; Fig. 8B, Drosha2, D2, siRNA). Quantitation of this and other data show that the relative amount of the mgU2-30 fragment normalized to full-length scaRNA9 is reduced 60% for Drosha2 siRNA and 70% for Drosha4 siRNA compared to control siRNA (Fig. 8C).

We next examined if Drosha knockdown would alter the dynamics of endogenous scaRNA9 and scaRNA2. For this work, cells were transfected with control or Drosha2 siRNA for 48 or 72 h, followed by RNA isolation, northern blotting, and detection. The 72 h data is shown in Fig. 8D and E. For scaRNA9, Drosha reduction increased the amount of endogenous full-length scaRNA9, but did not impact the level of processed mgU2-30 fragment compared to negative control siRNA (Fig. 8D). The same membrane was reprobed for U3 snoRNA in order to verify that approximately equal amounts of RNA were loaded in each lane. Quantification of the full-length scaRNA9 signal normalized to the U3 snoRNA signal demonstrates that the relative amount of full-length scaRNA9 is significantly increased upon Drosha knockdown at both 48 and 72 h time points (Fig. 8F). In contrast, the amount of endogenous full-length scaRNA2 is not obviously affected by Drosha knockdown after 72 h (Fig. 8E). These findings indicate that Drosha may impact the processing of full-length scaRNA9 into the mgU2-30 fragment.

## DISCUSSION

An exciting emerging concept is that of ribosome heterogeneity, leading to specialized ribosomes. This concept is based on the realization that ribosomes are not all the same, all the time, but can vary in response to different physiological or pathological situations (Lafontaine, 2015). By having specialized ribosomes, the cell is able to increase or decrease the translation of certain mRNAs, resulting in a protein composition that is optimized for a given environment. There are approximately 200 modifications in human rRNA (around 100 each of pseudouridylation and ribose methylation), the majority of which are mediated by snoRNPs (Lafontaine, 2015). The long-term goal of research in the rRNA field is to understand how each of these modifications contribute to translation. With our present tools and expertise, the scientific community is unable to decipher the role of individual rRNA modifications on translation. However, studies into how each modification site within rRNA is regulated is possible with current technology, and it may be that regulatory RNPs (Poole et al., 2017) are important players in this regulation. We have recently reported that regulatory RNPs may influence that modification of 18S rRNA, 28S rRNA and U6 snRNA, possibly by interactions with the snoRNPs snord16, snord68, snord111 and snord94 (Burke et al., 2018; Poole et al., 2017). Since the nucleolus-enriched fragments derived from scaRNA 2, 9 and 17 may form regulatory RNPs that influence rRNA modifications by altering snoRNP activity, it is possible that the processing of scaRNA 2, 9 and 17 could change in response to environmental conditions. In so doing, the processing of scaRNA 2, 9 and 17, which changes the ratio of full-length scaRNA (enriched in the CB) to processed fragment (enriched in the nucleolus) may be part of an overall cell response required to adjust to environmental conditions by forming specialized ribosomes. The data presented here clearly support this hypothesis, given that six different stress conditions (etoposide, cisplatin, heat shock, oxidative stress, serum starvation and serum reduction) all alter the full-length scaRNA to processed mgU2-30 fragment ratio of ectopically expressed scaRNA9 (Figs 2 and 3). Furthermore, examination of endogenous scaRNA9 and scaRNA2 shows that the amount of these full-length scaRNAs is increased after etoposide treatment (Fig. 3F,G).

The data presented in Figs 2 and 3 are consistent with the idea that the various treatments impact the processing of full-length scaRNA 9 and 2, which results in the formation of the nucleolus enriched mgU2-30 and mgU2-61 fragments, respectively. Additionally, and/or alternatively, the treatments used here may be altering the overall transcription levels of the scaRNA9 host gene or the scaRNA2 gene, which is independently transcribed. Yet another possibility is that these treatments are altering the degradation of full-length scaRNA2 and scaRNA9 or degradation of the fragments derived from these scaRNAs. In regard to endogenous scaRNA9, we show that etoposide treatment does not have a noticeable impact on mgU2-30 fragment levels, but full-length scaRNA9 is increased (Fig. 3F). Likewise, Drosha reduction increases endogenous full-length scaRNA9, but mg-U2-30 levels are not affected (Fig. 8D). Both results are consistent with a decrease in the processing of full-length scaRNA9 to the mgU2-30 fragment since mgU2-30 fragment levels did not change. A further complexity to consider in regards to the dynamics that regulate the ratio of the processed fragments to full-length scaRNA 2, 9 and 17 is that these fragments may, in fact, not be derived from full-length scaRNA 2, 9 and 17 at all. Instead, it is possible that these fragments (mgU2-61, mgU2-19, mgU2-30 and mgU4-8; Fig. 1) are generated directly from the primary transcripts (or debranched intron in the case of scaRNA9). In other words, some primary transcripts give rise to fragments whereas others form full-length scaRNA 2, 9 or 17. More work will be needed to further clarify the mechanisms that regulate the levels of full-length scaRNA 2, 9 and 17 and the processed fragments mgU2-61, mgU2-19, mgU2-30 and mgU4-8.

Given that several studies have shown that many of the stress conditions used here disrupt nuclear organization, including CB composition and coilin localization (Hebert, 2010), we next examined if alterations in CB composition correlated with changes in scaRNA 2 and 9. This work is especially justified considering that the endogenous coilin interactome is highly enriched for scaRNA2, scaRNA9 and scaRNA17 (Enwerem et al., 2014). Our analysis showed that etoposide and cisplatin disrupted CBs, as reported previously (Broome and Hebert, 2013; Gilder et al., 2011; Song et al., 2017; Velma et al., 2012), but we observed that these disruptions did not alter the co-localization of coilin with WRAP53 (Fig. 4). In contrast, SMN often formed gems and was not co-localized as highly with coilin and WRAP53 upon etoposide or cisplatin treatment (Figs 5 and 6). These findings thus correlate the disruption of the coilin/WRAP53/SMN complex with alterations in the dynamics of scaRNA9 and scaRNA2. More studies will be needed to determine how the coilin/WRAP53/SMN complex facilitates scaRNA9, scaRNA2 and possibly scaRNA17 processing, but our previous work has shown that reduction in the levels of these proteins does indeed alter scaRNA processing (Enwerem et al., 2015). Specifically, we have found that the reduction of coilin is associated with increased scaRNA2 processing while SMN reduction is associated with reduced scaRNA2 processing (Enwerem et al., 2015). SMN, therefore, may be a positive contributor towards scaRNA processing. This hypothesis is supported by our SMN knockdown and rescue experimental results in Fig. 7, which show that SMN does indeed positively contribute to the processing of scaRNA9. Future work will examine the role of SMN in scaRNA processing in more detail. For example, it would be of interest to determine if SMN mutations found in SMA patients disrupt the putative function of SMN in scaRNP biogenesis. In addition to studies with SMN, future work will also examine the role of coilp1 in the processing of scaRNA 2, 9 and 17. Coilp1 is derived from a coilin pseudogene, and may have functional redundancy with coilin, in addition to its own activities (Poole et al., 2016).

Collectively, the work presented here strongly suggests that the dynamics of scaRNA9 and scaRNA2 are regulated and may be part of a signaling system that seeks to optimize rRNA modifications and yield ribosomes most suited to generate a constellation of proteins that can best manage the stress condition. Future work in this area would be greatly facilitated by a comprehensive assessment of how rRNA modifications change in response to the stress conditions used here. In addition to scaRNA9 and scaRNA2, it is likely that scaRNA17 is also subjected to regulatory events that control the ratio of mgU4-8 to full-length scaRNA17, but this has not been proven. We are currently examining scaRNA17 dynamics with the treatments and conditions used here, as well as evaluating other conditions and incubation times to determine if these alter scaRNA2, scaRNA9 and scaRNA17 processing. It is possible that, since scaRNA9 is subjected to two processing events, generating mgU2-19 and mgU2-30, disruptions in processing are more easily detected for this scaRNA compared to scaRNA 2 and 17. Alternatively, since scaRNA 2 and 17 are independently transcribed, their processing may be subjected to other controls compared to the intron-encoded scaRNA9. Hence, our current efforts are exploring the mechanisms by which scaRNA 2 and 17 processing is regulated and determining if these pathways are the same as used for the intron-encoded scaRNA9. Our finding that Drosha may be involved in controlling the ratio of fragment to full-length scaRNA 2 and 9 is especially interesting considering the direct ties of Drosha and Dicer to the DNA damage response (Francia et al., 2012), and the similar impact of DNA damaging agents (Figs 2 and 3) and Drosha knockdown (Fig. 8) on scaRNA 2 and 9 dynamics. We have previously reported that coilin participates in the suppression of RNA polymerase I in response to cisplatin-induced DNA damage (Gilder et al., 2011). This finding, along with the data presented here, strongly argue that the CB and components thereof are responsive to environmental conditions and play an active role in nucleolar activity, including ribosome biogenesis.

## MATERIALS AND METHODS

### Cell lines, plasmids, transfections and treatments

HeLa cells were obtained from the American Type Culture Collection (ATCC) and cultured as previously described (Poole et al., 2016). Ectopic expression of scaRNA2, scaRNA9 and scaRNA17 was achieved using the pcDNA3.1+ expression vector, as previously described (Enwerem et al., 2014, 2015; Poole et al., 2017). EGFP-C1 (empty GFP vector encoding GFP only) and GFP-SMN were described previously (Poole and Hebert, 2016). DNA transfections were conducted using FuGene HD (Promega, Madison, USA) according to the manufacturer's protocol. For siRNA transfections, RNAiMax was utilized (Invitrogen). Negative control SMN siRNAs were ordered from Integrated DNA Technologies (Coralville, USA). Two different SMN siRNAs were used: SMNA, forward (5′- CCACUAAAGAAACGAUCAGACAGAT-3′), reverse (5′-AUCUGUCUGAUCGUUUCUUUAGUGGUG-3′); SMNB, forward (5′-CCACUAAAGAAACGAUCAGACAGAT-3′), reverse (5′- AUCUGUCUGAUCGUUUCUUUAGUGGUG-3′). Two different Drosha siRNAs were used: DROSHA 2, forward (5′-AAUCAGGAUUGGAAUGACCCCAAAT-3′), reverse (5′-AUUUGGGGUCAUUCCAAUCCUGAUUCA-3′); DROSHA 4, forward (5′-CAACUGUUAUAGAAUACGAUGAUCA-3′), reverse (5′-UGAUCAUCGUAUUCUAUAACAGUUGGC-3′). For experiments in which cells were transfected with DNA after siRNA treatment, siRNA treatment was for 24 h, followed by DNA transfection and incubation for an additional 24 h. At harvest, therefore, these cells were subjected to 48 h of siRNA treatment and 24 h of plasmid expression.

For cell treatments, HeLa cells were grown at 37°C in DMEM (Dulbecco's Modification of Eagle's Medium) containing glucose, glutamine and sodium pyruvate supplemented with 10% fetal bovine serum until 70% confluent and then transfected with pcDNA3.1+scaRNA9 for 7 h. The cells were then subjected to the following treatments for 17 h (resulting in 24 h total DNA expression): 3 μg/ml cisplatin, 7.5 μM etoposide, 42°C incubation, 0.2 mM H_2_O_2_, DMEM without serum or incubation in Optimem reduced serum medium (Invitrogen).

### Northern blotting

RNA was harvested using TRI-Reagent (Sigma-Aldrich) following the manufacturer's suggested protocol. 10 μg of RNA was run on a 6% denaturing polyacrylamide gel (Invitrogen) in 1X Tris-Borate-EDTA (TBE) at 200 V for 32 min. The gel was then washed in 1X TBE for 10 min with gentle shaking. The RNA was then transferred onto a positively charged nylon membrane (Invitrogen) with the iBlot Gel Transfer device (Life Technologies, Grant Island, USA) using program 5 for 5 min or program 5 for 10 min (for endogenous). After transfer, the membrane was rinsed in ultrapure water, allowed to dry, and then subjected to a UV cross-linker (UVP, Upland, USA) at a setting of 120,000 μJ/cm^2^. The membrane was then placed in a hybridization bottle and pre-hybridized using 15 ml of Ultrahyb Ultrasensitive Hybridization buffer (Ambion; Life Technologies) for 30 min at 42°C in a hybridization oven. The DNA oligo probes used to detect scaRNA9 were a 5′ DIG probe as previously described (Poole et al., 2017), a 5′ and 3′ DIG probe (5′-TAGAAACCATCATAGTTACAAAGATCAGTAGTAAAACCTTTTCATCATTGCCC-3′), or a 3′ DIG labeled probe (Poole et al., 2016) created using the DIG Oligonucleotide Tailing Kit, 2nd Generation (Roche, Indianapolis, USA) according to the manufacturer's protocol. Membranes were then prepared for detection using the DIG Wash and Block kit (Invitrogen) following the manufacturer's suggested protocol with the Anti-DIG antibody used at 1:10,000. Detection was carried out using CSPD (Roche, Mannheim, Germany) following the manufacturer's suggested protocol. Blots were imaged using a Chemidoc imager (Bio-Rad), Adjustments to images were made using the transformation settings on QuantityOne software and applied across the entire image.

### Western blotting

HeLa cells were transfected with negative control or SMNB siRNA for 24 h and then co-transfected with scaRNA9 pcDNA 3.1+ and GFP empty vector or GFP-SMN for another 24 h. For Drosha knockdown, HeLa cells were transfected with negative control, or DROSHA 2 siRNA for 48 h. Protein was harvested as previously described (Poole et al., 2016). 15 μl of lysate was run on a precast 10% Mini-Protean Gel (Bio-Rad). Western transfer and detection was then conducted as previously described (Poole et al., 2016). The primary antibodies used were: anti-GFP mouse mAb (Roche), anti-SMN mouse mAb (BD Biosciences, San Jose, USA), anti-β-tubulin mouse mAb (Sigma-Aldrich), and anti-Drosha rabbit mAb (Cell Signaling). Secondary antibodies used were goat anti-mouse HRP and goat anti-rabbit HRP. Bands were detected with SuperSignal West Pico Chemiluminescent Substrate (Thermo Fisher Scientific) following the manufacturer's suggested protocol and imaging was done on a ChemiDoc (Bio-Rad) with QuantityOne software. Adjustments to images were made using the transformation settings on QuantityOne software and applied across the entire image.

### Immunofluorescence

HeLa cells were grown on 8-well or 4-well glass slides. Each well was treated individually with 9 μM etoposide, 17 μM etoposide, or 3 μg/μl cisplatin for 24 h. Cells were fixed in 4% paraformaldehyde (PFA) for 10 min, permeabilized in 1X phosphate-buffered saline (PBS) containing 0.5% Triton for 5 min, and then rinsed with 1X PBS three times. Slides were blocked in 10% normal goat serum (NGS) at 37°C for 30 min. Slides were then probed with 1:200 anti-coilin mouse monoclonal antibody (Santa Cruz Biotechnology), 1:200 anti-coilin rabbit polyclonal antibody (Santa Cruz Biotechnology),1:100 anti-SMN mouse monoclonal antibody (BD Biosciences), or 1:200 anti-WRAP53 rabbit monoclonal antibody (Bethyl Laboratories, Mongomery, USA) in 10% NGS at 37°C for 30 min. Slides were then washed with 1X PBS for 5 min three times and incubated with 1:600 Alexa Fluor 594 (A11012, Invitrogen) goat anti-mouse (red) or 1:600 Alexa Fluor 488 (A11001, Invitrogen) goat anti-rabbit (green) secondary antibody in 10% NGS at 37°C for 30 min. Slides were then washed three times in 1X PBS for 5 min each wash, and then 4′,6-diamidino-2-phenylindole (DAPI) stained to detect the nucleus followed by coverslip mounting with Antifade (Invitrogen). Images were captured on a Nikon Eclipse E600 epiflourescence microscope, and digital images were taken using Photometics CoolSnap HQ2 CCD camera and processed using MetaView software. PowerPoint and Adobe Photoshop Elements 7 were used in the preparation of images, as previously described (Poole et al., 2016).
